# Numerical investigation of low-frequency shock train oscillations in a divergent isolator with vortex generator jets

**DOI:** 10.1371/journal.pone.0328630

**Published:** 2025-07-17

**Authors:** Jinlong Wang, Kaijie He, Qingchun Zhou, Jinli Wang

**Affiliations:** 1 Chengde College of Applied Technology, Chengde, P. R. China; 2 School of Aerospace Engineering, Huazhong University of Science and Technology, Wuhan, P. R. China.; NED University of Engineering and Technology, PAKISTAN

## Abstract

This study numerically investigates the low-frequency oscillations of shock trains within a two-stage divergent isolator equipped with four vortex generator jets, operating at Mach 3.034 under a constant backpressure of 0.25 MPa. Detailed flow field analysis and spectral examination of pressure signals provide a comprehensive quantitative and qualitative understanding of the unsteady behavior and its underlying mechanisms. The results reveal that the shock train undergoes low-frequency streamwise oscillations at 102 Hz, with displacement amplitudes reaching up to 2.5 times the isolator inlet height. This oscillatory behavior is characterized by a spring-like effect within the shock train and a wave-like breathing effect in the separation region. Two distinct driving mechanisms govern the oscillatory behavior of the shock train, resulting in characteristic path independence for its upstream and downstream motions. The dominant mechanism is attributed to the inherent instability of the separation region (downstream mechanism), while spatial non-uniformities introduced by the upstream vortex generator jets and the duct geometry act as secondary amplifying factors, collectively contributing to the oscillatory behavior.

## 1. Introduction

**Fig 1:**
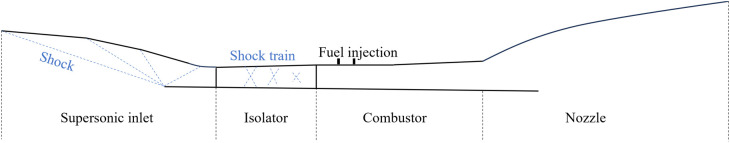
Schematic diagram of a scramjet.

With the obvious advantages of structural simplicity and high specific impulse, the scramjet is considered one of the most promising propulsion systems for future hypersonic missions [1–3]. As illustrated in Fig 1, a typical scramjet comprises an inlet, isolator, combustor, and nozzle. The isolator, located between the inlet and the combustor, is often a constant-area or slightly divergent duct. It plays a key role in balancing the pressure rise caused by combustion and preventing the interaction between the upstream compression and downstream combustion process [4,5]. The pressure balance is maintained by a shock train, a complex shock wave-boundary layer interaction (SWBLI) phenomenon [6]. The SWBLI is inherently complex, often accompanied by viscous features such as boundary layer separation, shear layers, recirculation bubbles, and vortex shedding [7–10].

In recent years, significant progress has been made in shock train research through theoretical, numerical, and experimental approaches, particularly in the areas of structural characteristics [4,11,12], shock train model [13,14], dynamic behavior [15,16], and flow control methodologies [17–19]. Gnani et al. reviewed the results of previous studies in 2016 [20]. The behavior of the shock train is influenced by various factors, including inflow conditions [21,22], duct geometry [23,24], backpressure [25], wall thermal state [26], boundary layer characteristics [27], and flow control measures.

Despite extensive research, the mechanisms underlying low-frequency self-excited oscillations of the isolator shock train remain poorly understood. Studies have shown that even when external parameters are held constant, shock trains can exhibit low-frequency self-excited oscillations at their time-mean positions [16,28], with displacements up to three times the height of the duct. These oscillations occur at frequencies one to two orders of magnitude lower than the characteristic frequency of the turbulent boundary layer [29,30]. Such unsteady behaviors can seriously degrade engine performance, leading to structural fatigue, thermal loading fluctuations, noise, combustion instability, and potential inlet unstart.

Two primary hypotheses have been proposed regarding the cause of such low-frequency oscillations: (1) the upstream mechanism, attributing the instability to fluctuations in the incoming boundary layer, and (2) the downstream mechanism, which links the oscillation to instabilities in the separation region. Humble et al. [31] identified hairpin vortex formation with low-momentum regions (0.5 correlation between velocity fluctuations and separation location), consistent with Wu et al.’s [32] findings of comparable coefficients. Priebe et al. [33] demonstrated that low-frequency shock motion predominantly correlates with separated bubble dynamics and shear layer flapping, with minimal statistical influence from the inflow boundary layer. This downstream dominance was reinforced by Sugiyama et al. [34], who identified oscillation origins in the primary shock’s separation zone. Subsequent investigations by Wu [32], Souverein [35], and Clemens [36] established a scale-dependent consensus: large-scale motions (>4δ) stem from downstream separation instabilities, while smaller scales (<2δ) originate from upstream boundary layer fluctuations. A comprehensive review by Clemens and Narayanaswamy [37] concluded that downstream mechanisms dominate strongly separated flows, with combined mechanisms emerging only in weakly separated cases.

However, most previous studies are based on simplified or quasi-two-dimensional geometries. In practical applications, isolators often employ three-dimensional divergent geometries and integrate active flow control devices such as vortex generator jets (VGJs) to enhance backpressure tolerance [38]. The introduction of such three-dimensional non-uniform disturbances significantly complicates the shock train dynamics. Consequently, a research gap exists concerning the behavior and mechanism of low-frequency self-excited oscillations in slightly divergent rectangular isolators with VGJs.

The present paper investigates the low-frequency oscillation behavior and mechanism of shock trains in a two-stage divergent isolator with vortex generator jets, using computational fluid dynamics (CFD). This study addresses two key scientific questions concerning the oscillation characteristics and driving mechanisms, providing theoretical guidance for isolator design and flow control strategies in high-speed internal flow applications, such as over-expanded nozzles, supersonic combustors, and supersonic inlets.

The remainder of this paper is organized as follows: Section 2 introduces the numerical methodology, isolator model, and validation. Section 3 analyzes the low-frequency oscillation behavior of the shock train in the isolator with VGJs. In Section 4, the underlying oscillation mechanisms are investigated through flow field and spectral analysis. Key conclusions are summarized in Section 5.

## 2. Methods

### 2.1. Numerical method and verification

The three-dimensional compressible Reynolds-averaged Navier-Stokes (RANS) equations are solved by using the commercial software ANSYS Fluent with double precision accuracy. The RANS simulations were conducted in an unsteady state by utilizing the Shear Stress Transport (SST) *k-ω* turbulence model, which has been demonstrated to accurately predict the complex SWBLI flow structures in previous works [39–41]. The simulations were performed utilizing a density-based solver and the Roe-FDS flux type. The flow term employed a second-order upwind scheme for spatial discretization. During the steady-state initialization stage, the first-order upwind scheme was employed for the spatial discretization of the turbulent kinetic energy and specific dissipation rate terms. Upon convergence of the solution, the scheme was switched to a second-order formulation for the subsequent unsteady simulations. To advance the equations in time, a second-order implicit method was chosen for the transient formulation.

The governing equations, including continuity, momentum, and energy equations, are as follows:


$$\frac{\partial \rho }{\partial t}+\nabla \cdot \left(\rho \vec{v}\right)=0$$ (1)

$$\frac{\partial }{\partial t}\left(\rho u_{i}\right)+\frac{\partial }{\partial x_{j}}\left(\rho u_{i}u_{j}\right)=-\frac{\partial p}{\partial x_{j}}+\frac{\partial \tau_{ij}}{\partial x_{j}}$$ (2)

$$\frac{\partial }{\partial t}\left(\rho E\right)+\frac{\partial }{\partial x_{j}}\left(\rho h_{t}u_{j}\right)=\frac{\partial }{\partial x_{j}}\left(u_{i}\tau_{ij}+\lambda \frac{\partial T}{\partial x_{j}}\right)$$ (3)

*𝜏*_*ij*_ is the shear stress tensor, defined as,


$$\tau_{ij}=\mu_{eff}\left(\frac{\partial u_{i}}{\partial x_{j}}+\frac{\partial u_{j}}{\partial x_{i}}-\frac{2}{3}\delta_{ij}\frac{\partial u_{k}}{\partial x_{k}}\right)$$ (4)

The transport equations for calculating turbulence kinetic energy (*k*) and the specific dissipation rate (*ɷ*) are as follows,


$$\frac{\partial (\rho k)}{\partial t}+\frac{\partial \left(\rho ku_{i}\right)}{\partial x_{i}}=\frac{\partial }{\partial x_{j}}\left[\left(\mu +\frac{\mu_{t}}{\sigma_{k}}\right)\frac{\partial k}{\partial x_{j}}\right]+G_{k}-\Delta_{k}$$ (5)

$$\frac{\partial (\rho \omega )}{\partial t}+\frac{\partial \left(\rho \omega u_{i}\right)}{\partial x_{i}}=\frac{\partial }{\partial x_{j}}\left[\left(\mu +\frac{\mu_{t}}{\sigma_{\omega }}\right)\frac{\partial \omega }{\partial x_{j}}\right]+G_{\omega }-\Delta_{\omega }+D_{\omega }$$ (6)

The fluid was considered to be single-species thermally perfect air. The specific heat and viscosity were computed using the piecewise-polynomial method and Sutherland’s viscosity law, respectively, which is as follows,


$$\mu =\mu_{ref}\frac{T_{ref}+S}{T+S}{\left(\frac{T}{T_{ref}}\right)}^{1.5}$$ (7)

**Fig 2:**
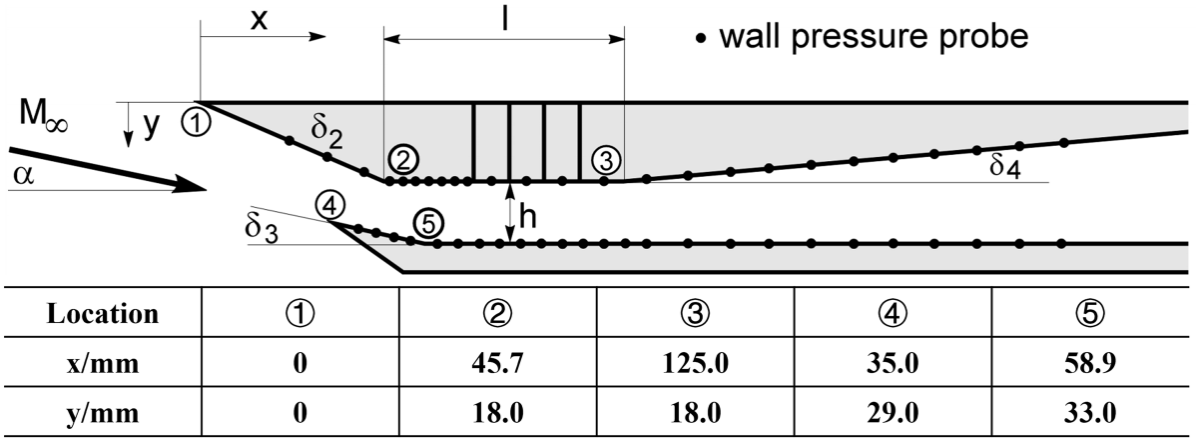
Geometric parameters of the model [42].

The numerical method was validated by comparing its results with experimental data from Reference (42). The reference provided detailed geometric parameters and operating conditions of the isolator. In this study, numerical simulations were conducted for the 79.3 mm isolator structure, with the geometric parameters and coordinates illustrated in Fig 2. The freestream Mach number was set to *Ma*_∞_=2.5, and the throttling degree Δ = 0% (fully opened).

A fully structured grid was employed, with a total grid number of 162,668 cells. Near-wall grids were refined, with the first layer thickness set to 0.005 mm and a growth rate of 1.15, ensuring a Y+ value below 1.

**Fig 3:**
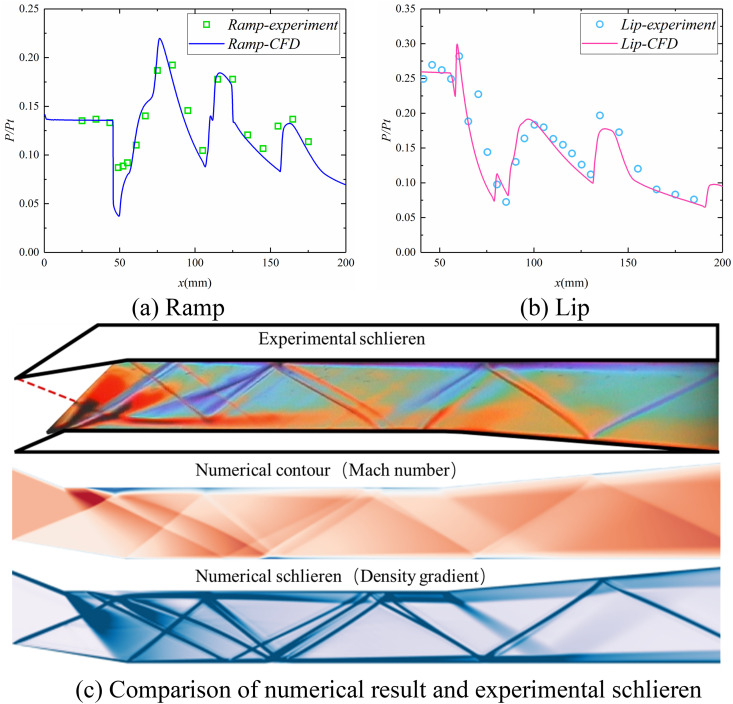
Comparison of numerical and experimental results: (a) Ramp; (b) Lip; (c) Schlieren.

As shown in Fig 3(a-b), the pressure distribution along the wall exhibits a consistent trend with the experimental pressure measurements. The intricate flow features observed in the experimental schlieren images, including the expansion and separation zones at the ramp turning point, as well as the reflection of shock waves and expansion waves within the isolator, are effectively replicated in the numerical results (Fig 3(c)). This demonstrates that the numerical method employed in this study can accurately capture the complex flow field within the isolator, providing robust support for the subsequent research.

### 2.2. Isolator model and grid convergence

**Fig 4:**
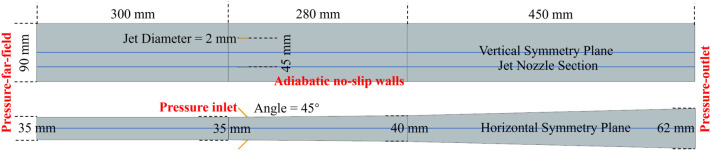
The isolator schematic diagram and boundary conditions.

The isolator model in this paper is a two-stage divergent rectangular duct, with the height of the inlet and outlet cross-sections of the first divergent segment being 35 mm and 40 mm, respectively, and a length of 280 mm. The height of the outlet of the isolator is 62 mm, with a length of 450 mm. The walls on both sides are parallel structures with a width of 90 mm. Four 2 mm-diameter jet pipes, angled at a 45–degree pitch, are installed at a distance of 30 mm from the entrance of the isolator, and at the 1/4 and 3/4 positions in the spanwise direction, with a total jet pressure of 2 MPa. This study focuses on the flight condition corresponding to a Mach number of 6.0 at an altitude of 26 km, yielding a compression ratio of 29.9 for the ramjet inlet. The flow conditions at the inlet of the isolator (the outlet of the supersonic inlet) are summarized in Table 1, with a Mach number of 3.034, a static temperature of 651.5 K, and a static pressure of 65,446 Pa. The backpressure of the isolator is 0.25 MPa. All walls are assumed to be adiabatic and non-slip. A 300 mm-long straight section is installed in front of the isolator to develop the boundary layer for approaching the actual operating conditions. The detailed schematic diagram and boundary conditions are shown in Fig 4 and Table 1, respectively.

**Table 1:** Incoming parameters and backpressure.

Mach number	Temperature/K	Pressure/Pa	Backpressure/MPa
3.034	651.5	65446	0.25

**Fig 5:**
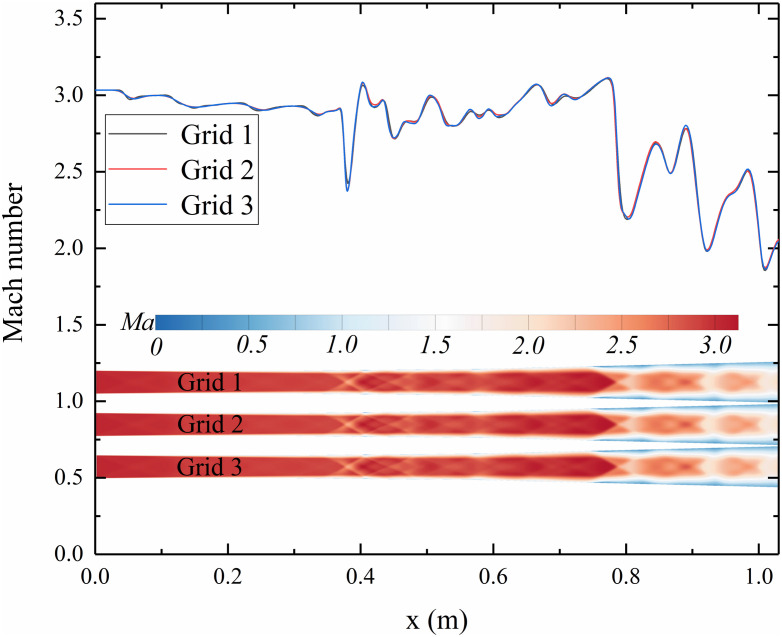
The Mach number distribution along the geometric centerline and vertical symmetry plane.

Three different grid resolutions were employed to validate grid independence, containing approximately 3.01 million (Grid 1), 4.52 million (Grid 2), and 6.78 million (Grid 3) cells, respectively. The height of the first grid layer was set to 0.01 mm to ensure that the Y⁺ values near the shock train regions remained below 1, and Y⁺ values along all walls are less than 5. Fig 5 shows the Mach number distributions along the geometric centerline and the vertical symmetry plane. It can be observed that the simulation results of all three grids show good qualitative agreement, especially in the shock train region. The major flow features, such as shock waves and separation zones, are consistent in both shape and location.

To quantitatively assess grid convergence, the Grid Convergence Index (GCI) method proposed by Roache [43] was employed. The total pressure recovery at the isolator exit, relative to the isolator inlet, was selected as the evaluation metric, with values of *f₁ = 0.352405*, *f₂ = 0.3524142*, and *f₃ = 0.3524146* for Grid 1 through Grid 3, respectively. A safety factor of 3 was adopted in the GCI calculations. A grid refinement ratio of *r = 1.5* was used, and the order of accuracy was set to *P = 2*. The GCI analysis demonstrates that the solution is within the asymptotic range, indicating that grid convergence has been achieved. Therefore, Grid 2 was chosen for all subsequent simulations to ensure both computational accuracy and efficiency.


GCI12=3×|(f1−f2)/(f1−f2)f2\nulldelimiterspacef2|/|(f1−f2)/(f1−f2)f2\nulldelimiterspacef2|(rp−1)\nulldelimiterspace(rp−1)≈0.006265% (8)

GCI23=3×|(f2−f3)/(f2−f3)f3\nulldelimiterspacef3|/|(f2−f3)/(f2−f3)f3\nulldelimiterspacef3|(rp−1)\nulldelimiterspace(rp−1)≈0.002724% (9)

$$r^{p}\times GCI_{23}\approx GCI_{12}$$ (10)

For the problem addressed in this study, further validation of grid error accumulation in unsteady simulations is essential. To estimate the unsteady error accumulation, the method proposed by Smirnov et al. [44] was adopted, which has been widely utilized in prior studies of shock train oscillations [45,46]. The integration of the relative errors for 3-D flow (*S*_*err*_), the maximal allowable number of time steps (*n*_*max*_), and the reliability ratio (*R*_*s*_) could be determined using the following formula:


$$S_{err}\approx \sum_{i=1}^{3}S_{i}\approx {\left(\frac{\Delta L}{L}\right)}^{P+1}+{\left(\frac{\Delta H}{H}\right)}^{P+1}+{\left(\frac{\Delta W}{W}\right)}^{P+1}$$ (11)

nmax=(Smax/SmaxSerr\nulldelimiterspaceSerr)2 (12)

RS=nmax/nmaxn\nulldelimiterspacen (13)

Here, *S*_*i*_ represents the mean ratio of cell size (*ΔL, ΔH, ΔW*) to the domain size (*L, H, W*) in the direction of integration. *P* = 2 indicates the use of second-order methods in the simulation. *S*_*max*_ denotes the allowable value of total error, with 5% chosen to calculate *n*_*max*_. In this study, a time step size of *Δt* = 1 × 10^−5^ s was employed, which is significantly smaller than the time required for air to traverse the isolator at freestream velocity. The accumulated error results are presented in Table 2, demonstrating the high reliability of the current simulation.

**Table 2:** Grid error accumulation in unsteady simulations.

Allowable error	Allowable number of time steps (*n*_*max*_)	Reliability
5%	6.18 × 10^9^	6.18 × 10^4^ (n = 1 × 10^5^, *t* = 1000 ms)
		1.24 × 10^3^ (n = 5 × 10^5^, *t* = 5000 ms)
		6.18 × 10^4^ (n = 1 × 10^6^,*t* = 10000 ms)

## 3. Results

**Fig 6:**
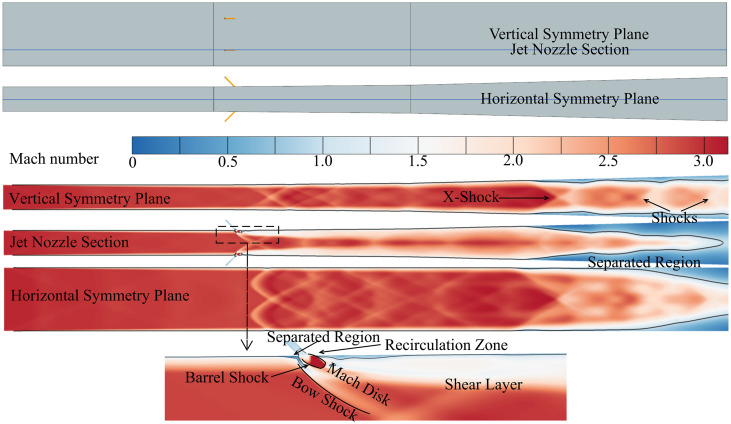
The primary flow field characteristics of the isolator.

The primary flow field characteristics under the current operating conditions, based on the steady-state results of the isolator shown in Fig 6, consist of two main components: the jet flow field and the shock train.

The high-pressure jet enters the isolator at a 45° angle and expands further into a supersonic state due to the pressure difference. A recirculation zone forms near the wall downstream of the jet as a result of the sudden cross-sectional expansion. The jet partially obstructs the supersonic airflow within the isolator, leading to the formation of a rearward-swept bow shock and a separation zone on the near-wall region ahead of the jet. The expanded supersonic main flow, being in an overexpanded state in the region behind the bow shock, forms Mach disks and oblique shock structures. The jet’s influence is limited in depth; its effect on the central flow in the duct is minimal. A shear layer forms between the region of reduced velocity downstream of the jet and the high-velocity main flow in the center of the duct.

Another prominent flow field characteristic is the shock train region downstream of the isolator. The shock train comprises a separation zone and multiple shock waves. The first shock exhibits an X-shock structure and is the strongest, while the subsequent shocks are relatively weaker. From the distribution of the separation zone on the symmetry plane of the isolator, small separation zones can be observed on the wall near the symmetry plane. These separation zones might represent parts of corner separation regions or may have been formed by localized boundary layer separation caused by the strong adverse pressure gradients induced by the shocks.

**Fig 7:**
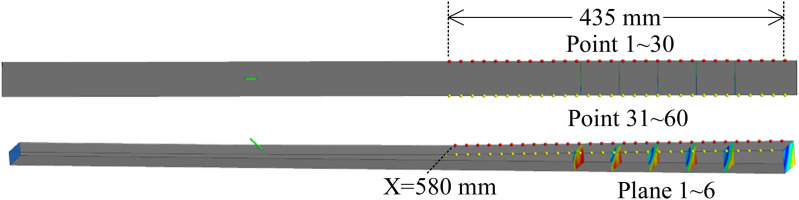
Cross-sections and monitoring points.

To monitor the intricate flow field within the shock train region, multiple transverse cross-sections and points were subjected to meticulous surveillance. The cross-sections are located at specific positions: *x* = 750 mm (the leading edge of the shock train), 800 mm, 850 mm, 900 mm, 950 mm, and 1030 mm (the outlet). Two sets of monitoring points are employed, situated along both the centerline and the diagonal line of the second divergent wall. Each set comprises 30 monitoring points, with a spacing of 15 mm between adjacent points, starting at the position of *x* = 580 mm. The detailed information is shown in Fig 7.

**Fig 8:**
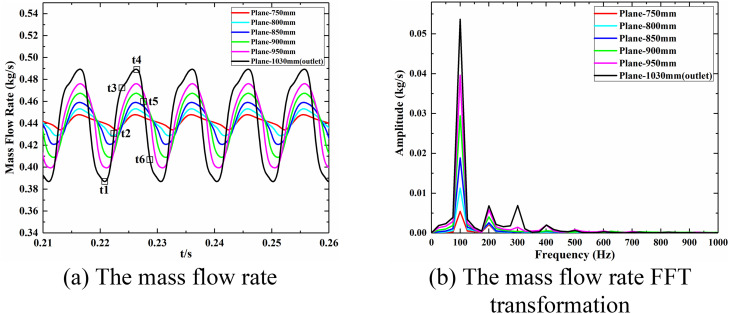
The mass flow rate and FFT transformation: (a) The mass flow rate; (b) FFT transformation.

The mass flow rates at planes 1–6 (as shown in Fig 7) within the shock train region were monitored, and the results are presented in Fig 8(a). The mass flow rates exhibit low-frequency periodic oscillations (S1 Table). Through FFT analysis, the dominant frequency of these oscillations was identified as 102 Hz, as shown in Fig 8(b). As the planes approach the exit of the isolator, the oscillation amplitude of the mass flow rate increases significantly. Additionally, the time-varying mass flow rate curves at different planes exhibit distinct waveforms and phase shifts. At the same plane, notable differences are observed between the rising and falling phases of the mass flow rate. For instance, at the isolator’s exit plane, the mass flow rate demonstrates a longer rising phase, while planes farther upstream display the opposite trend. This indicates that the self-excited oscillation in the shock train region is closely linked to changes in the morphological characteristics of the shock train.

**Fig 9:**
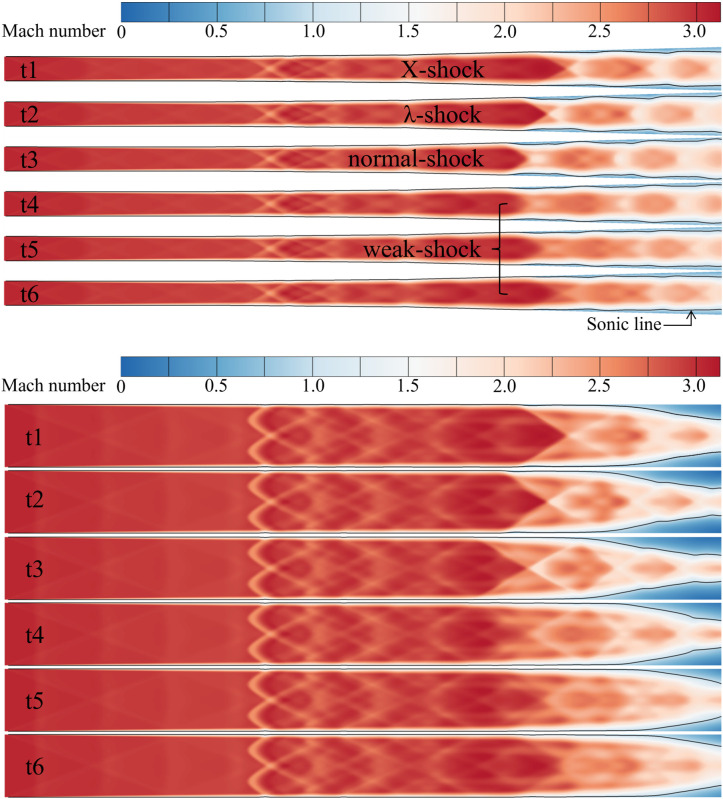
The Mach number distribution of the isolator.

Six specific moments within one oscillation cycle of the outlet mass flow rate curve are extracted for analysis. These moments include the curve trough (t1), rapid rise (t2), gradual rise (t3), curve peak (t4), rapid descent (t5), and gradual descent (t6), as indicated by the hollow points in Fig 8(a). The Mach number distributions on the symmetry plane at these moments are shown in Fig 9 (S1 Video). The self-excited oscillation of the shock train involves both an overall positional shift and relative morphological changes within the structure. Notably, the leading shock exhibits distinct transitions during the oscillation cycle. At t1, the leading shock takes the form of an X-shock. As the shock train moves forward at t2, the leading shock transitions into a λ-shock. By t3, as the forward movement continues, the shock angle increases, forming a curved shock structure. From t4, the shock train retreats toward the exit, with the leading shock becoming weaker and more diffuse. It is worth noting that on the vertical symmetry plane, the morphology of the leading shock shows minimal change from t1 to t3. During this phase, the separation zone is located far from the leading shock, gradually increasing in size and extending forward. In contrast, from t4 to t6, the leading shock weakens, and the separation zone diminishes in size. The self-excited oscillation flow field of the current shock train demonstrates two distinct flow characteristics during the upstream and downstream movement paths, highlighting the path-independent nature of the oscillation dynamics.

The position of the shock train’s leading edge plays a critical role in determining whether the engine operates normally. If the shock train’s leading edge enters the intake, it can lead to unstart conditions in the inlet. Therefore, analyzing the position variation of the shock train’s leading edge is essential from both the perspective of self-excited oscillation characteristics and isolator performance. In this study, three definitions for the shock train leading edge position are adopted.

**Fig 10:**
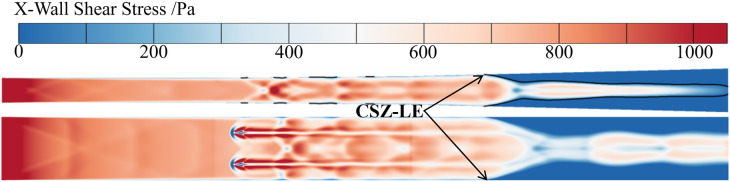
X.Wall Shear Stress on the wall.

Corner Separation Zone Leading Edge (CSZ-LE): Defined as the leading edge of the corner separation zone, identified through the streamwise shear stress distribution on the wall. This position corresponds to the location where the wall shear stress (X-wall shear stress) becomes zero, i.e., the transition point from positive to negative values, as shown in Fig 10. Pipe Centerline Leading Edge (PC-LE): Defined as the position of the first shock wave along the centerline of the pipe. Divergent Wall Leading Edge (DW-LE): Defined as the shock wave foot position along the centerline of the divergent wall. Since the shock waves do not directly interact with the wall, the identification of PC-LE and DW-LE positions relies on analyzing pressure gradients. These positions are determined as the points where the pressure gradient first exceeds 8 × 10^5^ kg ∙ m^-2^ ∙ s^-2^ along the pipe centerline and the divergent wall centerline, respectively.

**Fig 11:**
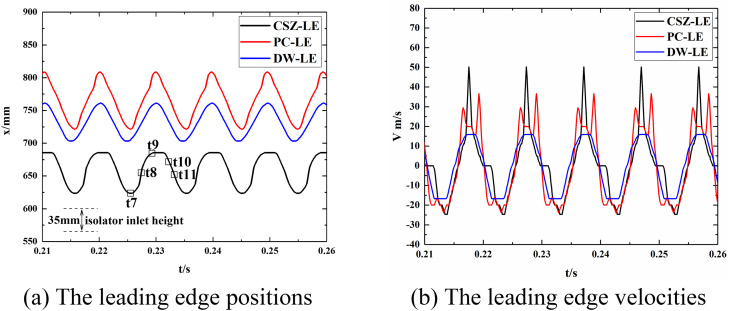
Positions and velocities at CSZ-LE, PC-LE, and DW-LE: (a) positions, and (b) velocities.

Fig 11 illustrates the temporal variation of the three leading edge positions and their velocities. The velocity is calculated as the slope of the position curve, with the positive direction aligned with the flow direction (S2 Table). The periodic motion of the shock train exhibits characteristics similar to a low-frequency sinusoidal pattern. The movement range of the shock train’s leading edge spans 1.65 to 2.5 times the height of the isolator inlet.

The maximum upstream and downstream velocities of the CSZ-LE are 24.69 m/s and 50.17 m/s, respectively. For the PC-LE, the corresponding velocities are 36.63 m/s and 23.89 m/s, while for the DW-LE, they are 15.92 m/s and 16.72 m/s. The PC-LE is influenced by the deformation of the shock from an X-shock to a λ-shock, resulting in velocity fluctuations. Notably, when the CSZ-LE reaches its most downstream position, it experiences a pause lasting for 0.193 of a complete oscillation period. During this time, both the PC-LE and DW-LE begin moving upstream. This observation suggests an increase in the size of the separation zone and a corresponding increase in the angle of the separation shock wave during this phase.

## 4. Discussion

By monitoring pressure levels at various positions and conducting correlation analysis, it becomes possible to quantify the individual contributions of each location to the unsteady oscillations exhibited by the shock train. Thus, this approach facilitates a deeper comprehension of the fundamental mechanisms that drive low-frequency oscillations. This approach has demonstrated its applicability in many studies focused on oscillation source identification [47]. FFT is applied to the pressure signals obtained from the monitoring points, yielding coherence and phase. The coherence measures the degree of similarity between two time signals, and the phase quantifies the temporal shift. The phase in the present paper is expressed in terms of angles and exhibits a relative nature. The definitions of coherence and phase between two sets of time signals *α*(t) and *β*(t) are as follows:


$$C_{\alpha \beta }\left(f\right)=\frac{{\left|P_{\alpha \beta }\left(f\right)\right|}^{2}}{P_{\alpha \alpha }\left(f\right)P_{\alpha \alpha }\left(f\right)}$$ (14)

$$\theta_{\alpha \beta }\left(f\right)=\arctan\frac{Q_{\alpha \beta }\left(f\right)}{G_{\alpha \beta }\left(f\right)}$$ (15)

where *P*_αα_(f) and *P*_ββ_(f) denote the self-PSD of signals *α*(t) and *β*(t), and *P*_αβ_(f) denotes the cross-PSD between signals *α*(t) and *β*(t). *G*_αβ_(f) and *Q*_αβ_(f) are the real and imaginary parts of *P*_αβ_(f), respectively. *P*_αβ_(f) can be expressed as


$$P_{\alpha \beta }\left(f\right)=G_{\alpha \beta }\left(f\right)-jQ_{\alpha \beta }\left(f\right)=\left|P_{\alpha \beta }\left(f\right)\right|e^{-jQ_{\alpha \beta }\left(f\right)}$$ (16)

**Fig 12:**
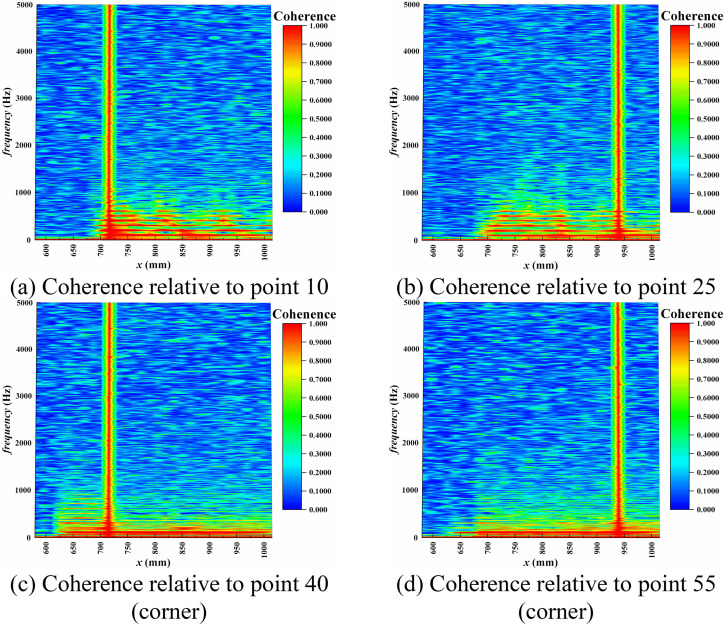
Coherence between the pressure at points 10, 25, 40, and 55.

**Fig 13:**
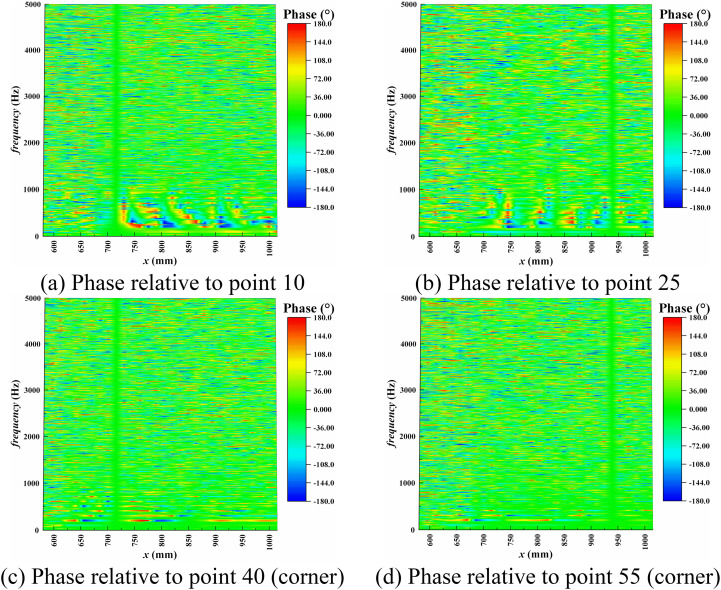
Phase between the pressure at points 10, 25, 40, and 55.

The pressure time signals from four monitoring points, namely points 10, 25, 40, and 55, were selected as references. The specific positions of these monitoring points have been indicated in Fig 8, which correspond to the signals from the intermediate and corner regions of the shock train leading edge and trailing edge areas, respectively. Figs 12 and 13 provide the spectra distribution of coherence and phase concerning these four monitoring points.

In the shock train region, there is a clear high correlation in the 102 Hz dominant frequency range and its harmonics, whereas no such correlated regions were observed upstream of the shock train. Among these, the highly correlated region at the corner measurement points extends to the very leading edge of the separation zone, while the highly correlated region at the mid-wall measurement points is relatively smaller in scope. This corresponds well to the leading edge positions of the shock angle and the separation zone, offering a new approach for dynamic monitoring of the shock train’s leading-edge position.

Furthermore, in the low-frequency range, as depicted in Fig 13, the phase in the intermediate region differs significantly from that in the corner region. The phase differences in the intermediate region suggest that the internal relative motion and shock deformation during the shock train’s oscillation process lead to delayed pressure signals in this region. In contrast, the phase differences in the corner separation region exhibit remarkable stability, maintaining a consistent and fixed phase difference at regular intervals along the streamwise direction. The pressure signals in the corner separation region are minimally affected and display pronounced linear regularity along the flow direction. They behave more like a global perturbation source that remains independent of specific locations.

Hence, the low-frequency self-excited oscillations of the shock train in this case are driven by a downstream mechanism—namely, the unstable separation zone drives the self-excited oscillatory behavior of the shock train.

The periodic evolution of the flow field occurs exclusively within the shock train region, while the upstream flow field ahead of the shock train remains largely unaffected. The dominant low-frequency mode identified in the spectral analysis confirms the presence of a downstream-driven mechanism centered around the separation zone. However, the influence of upstream VGJs-induced disturbances on the self-excited oscillation of the shock train remains uncertain. To investigate this further, the temporal evolution of flow structures at several key moments within a single oscillation cycle is analyzed. By coupling frequency-domain observations with time-resolved flow field characteristics, the underlying physical mechanisms of shock train self-excited oscillation and its interaction with upstream VGJ disturbances are explored in detail.

**Fig 14:**
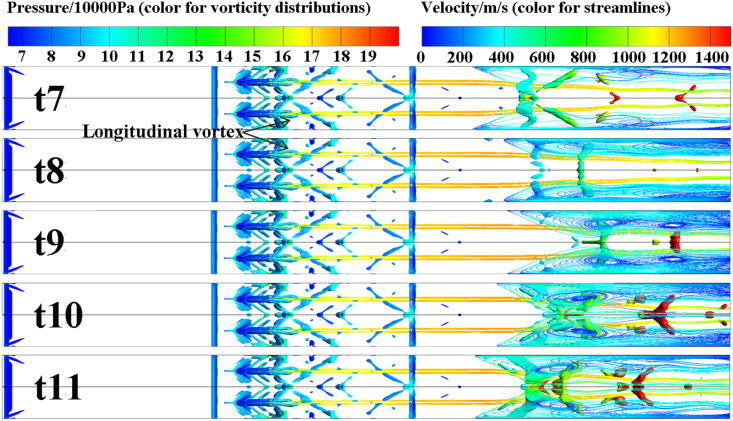
Streamlines (colored by velocity) and vorticity distributions (Q-criterion 3.38 × 10^8^ s^-2^, colored by pressure) at t7–t11.

**Fig 15:**
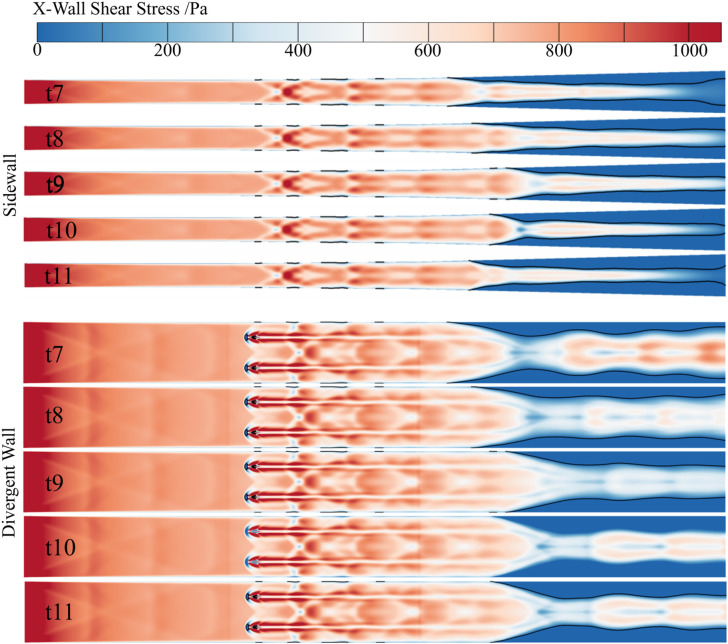
The X-Wall Shear Stress evolution on the wall at t7–t11.

**Fig 16:**
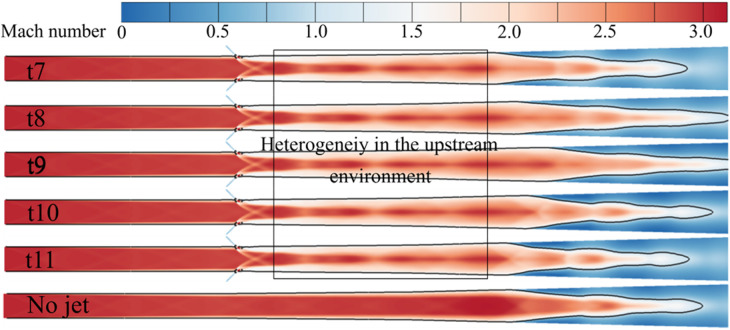
Mach number contours within the jet cross-section at t7–t11.

Five specific moments (t7 to t11), corresponding to five positions of the corner separation region’s leading edge within an oscillation cycle, are selected for analysis. These positions are marked in Fig 11(a), presented in the previous chapter. The flow streamlines and vorticity distributions at these moments (t7 to t11) during one oscillation period are shown in Fig 14. The wall shear stress distributions are depicted in Fig 15, where blue regions indicate negative shear stress values. The Mach number distributions within the jet cross-section are shown in Fig 16.

It can be observed that the size and shape of the separation zone exhibit significant variation during the shock train’s self-excited oscillation. When the shock train moves upstream, the size of the separation zone decreases, and the separation shock intensity increases due to a larger separation angle, which enhances the local pressure gradient. During upstream movement, influenced by the constricted cross-sectional area of the duct, the main flow Mach number decreases, reducing its resistance to adverse pressure gradients. Together, these factors, coupled with the separation zone’s instability, drive the shock train upstream. Due to the inertia of the subsonic separated flow in the boundary layer, the separation zone’s upstream movement tends to overshoot, weakening the restriction on the main flow. As a result, the intensity of the separation shock decreases, and the local adverse pressure gradient becomes insufficient to sustain further upstream motion of the separation zone. At this point, the main flow becomes the primary driving source, compressing the separation zone downstream. This leads to the shock train’s downstream movement.

During downstream movement, the downstream separation zone enlarges, driven by a larger separation angle. This intensifies the restriction on the flow area, causing the shock train to exhibit a state where the upstream shock weakens while the downstream shock strengthens. This behavior manifests as a “spring-like” effect in the shock train and a “wave-like breathing” effect in the separation region.

Furthermore, upstream jets induce the formation of vortex structures that draw high-energy fluid towards the near-wall region, creating a series of high-energy fluid streaks. This significantly enhances the kinetic energy reservoir in this region and improves its resistance to adverse pressure gradients. However, the non-uniform mixing of high- and low-energy fluids exacerbates flow instability within the separation zone. The jet effects cause the isolator flow field to exhibit pronounced three-dimensional asymmetric characteristics. This leads to the shock train’s leading edge encountering upstream environments with spatial heterogeneity at different positions. The modification of near-wall boundary layer characteristics induced by the jet, combined with the non-uniform distribution of the main flow, acts as a crucial trigger for the self-excited oscillation of the shock train.

**Fig 17:**
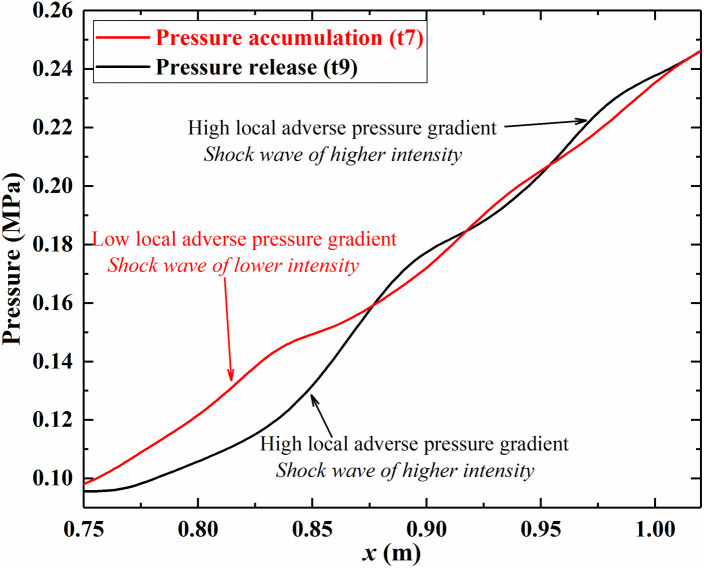
The pressure accumulation (t7) and release (t9) in the separation region.

The oscillation of the shock train essentially arises from the interaction between the unstable separation region and the main flow under adverse pressure gradients. Fig 17 presents the pressure distribution along the shock train region at two specific moments, t7 and t9, which correspond to two distinct states in the oscillation process. At t7, the separation shock at the leading edge of the shock train is weak, resulting in a relatively low adverse pressure gradient. During this phase, the main flow compresses the separation region, accumulating pressure within it. When the shock train retreats to t9, the separation region reaches its maximum size, exerting an even greater restriction on the main flow. This leads to stronger separation shocks and higher local pressures, particularly near the leading edge and close to the outlet. The high local adverse pressure gradient at the leading edge initiates upstream expansion of the separation region, releasing the accumulated pressure. The cyclic process of pressure release and accumulation in the separation region drives variations in the local adverse pressure gradient, which must match the boundary layer’s resistance to back pressure. This dynamic balance is the intrinsic essence underlying the self-excited oscillation of the shock train. It serves as the fundamental driving force for its motion and evolution.

Two distinct driving mechanisms work synergistically to govern the oscillation behavior of the shock train as it traverses distinct paths for upstream and downstream movements. This explains the path independence observed in the self-excited oscillation behavior of the shock train.

This study presents the first systematic investigation of low-frequency shock train oscillations in a three-dimensional divergent isolator with VGJs control. Unlike previous studies focused on two-dimensional or uncontrolled cases, the results identify a downstream-driven mechanism governed by separation zone instability and modulated by VGJs-induced spatial non-uniformities. The oscillation arises from a dynamic imbalance between adverse pressure gradients and boundary layer resistance, manifesting as a cyclic process of pressure accumulation and release. Moreover, the upstream and downstream movements of the shock train are driven by distinct mechanisms, leading to a path-independent oscillatory behavior—explained here for the first time through physical analysis. These findings uncover a coupled driving mechanism and provide new insight into shock train instability in complex flows, offering theoretical guidance for isolator design and active control strategies in high-speed propulsion systems.

## 5. Conclusions

This study numerically investigates the low-frequency oscillation behavior of a shock train in a two-stage divergent rectangular isolator influenced by vortex generator jets, aiming to explore the underlying mechanisms driving this phenomenon. Based on the analysis of the simulation results, the following conclusions have been drawn:

1. The shock train in the present case exhibits low-frequency oscillations with a dominant frequency of 102 Hz under constant backpressure, with a displacement amplitude reaching up to 2.5 times the inlet height of the isolator. This oscillatory behavior is accompanied by variations in shock strength and configuration, manifesting as a spring-like effect within the shock train and a wave-like breathing effect in the separation region.
2. Two distinct driving mechanisms govern the oscillatory behavior of the shock train, resulting in a characteristic path independence for its upstream and downstream motions. Upstream motion is driven by pressure release when the separation region is compressed by the main flow, while downstream motion occurs due to reduced local adverse pressure gradients when the separation region’s constraining effect on the main flow diminishes.
3. The low-frequency self-excited oscillations of the shock train are primarily governed by the inherent instability of the separation region (downstream mechanism). The spatial non-uniformity induced by upstream vortex generator jets and the duct geometry serve as secondary amplifying factors, collectively contributing to the oscillatory behavior.

The identified low-frequency oscillations, with a dominant 102 Hz and amplitudes up to 2.5 times the isolator inlet height, highlight potential structural and thermal risks in scramjet isolators under high backpressure. The path-independent nature of the oscillation, governed by alternating upstream and downstream mechanisms, suggests that steady-state models may underestimate dynamic loading effects. These insights emphasize the need for unsteady design considerations, especially during throttle transitions or off-design operation. While VGJs enhance backpressure tolerance, their induced spatial non-uniformities can amplify shock train instability. This underscores the importance of optimizing jet configuration not only for separation control but also for vibration suppression. The mechanisms revealed here offer guidance for robust isolator design and adaptive control strategies, and are extendable to other high-speed compressible flow systems such as supersonic combustors, over-expanded nozzles, and actively controlled inlets.

## Supporting information

S1 Table Mass flow rate periodic fluctuations at different cross-sections in the shock train region. Description: Contains time-resolved mass flow rate data at multiple cross-sections within the shock train region, used to characterize periodic flow fluctuations.
(XLSX)

S2 Table Temporal variation of the leading-edge position of the shock train. Description: Records the time-dependent position of the shock train leading edge, enabling calculation of its oscillation velocity.
(XLSX)

S1 Video Oscillation process of Mach number field in the symmetric plane of the shock train. Description: Dynamic visualization of Mach number field oscillations in the shock train symmetric plane.
(AVI)
